# Acute hepatitis as a manifestation of secondary syphilis: a case report and literature review

**DOI:** 10.11604/pamj.2024.48.44.42785

**Published:** 2024-06-05

**Authors:** Sakina El Mamouni, Maryeme Kadiri, Imane Ben El Barhdadi, Mohamed Borahma, Fatima-Zahra Chabib, Nawal Lagdali, Camelia Berhili, Fatima-Zahra Ajana

**Affiliations:** 1 Department of Hepato-Gastroenterology C, Ibn Sina Hospital, Mohamed V University, Rabat, Morocco

**Keywords:** Acute hepatitis, syphilis, cytolysis, case report

## Abstract

This is the case of a 25-year-old patient, with the notion of unprotected sexual relations with multiple partners consulted for cholestatic icterus with pruritus evolving for 2 months. The general examination found an intense mucocutaneous icterus. The examination of the lymph nodes revealed multiple lymph nodes. A thoracic-abdominal-pelvic scanner showed peri-portal edema and adenopathies above and below the diaphragm without suspicious lesions. Biologically, there was acute cytolysis with ASAT at 1612IU/L, ALAT at 1506IU/L, and icteric cholestasis, the acute viral serologies and other autoantibodies were all negative. Given the presence of adenopathy and sexual risk factors, a syphilis serology was requested and was positive: a TPHA at 2560UI/L, and a VDRL at 1/32 UI/L. A liver biopsy was performed, which showed the presence, on immunohistochemistry, of anti-treponemal-pallidum antibodies. After eliminating all etiologies of cytolytic hepatitis, we retained the diagnosis of syphilitic hepatitis. Therapeutically, we started a treatment based on ceftriaxone 2g/dl with spectacular biological improvement at H48 of the beginning of treatment.

## Introduction

Syphilis is a bacterial sexually transmitted infection caused by the spirochete treponema pallidum, first appeared in the late 15^th^ century and continues to be of great interest after more than five centuries. After almost disappearing in Western countries in the 1990s due to the prevention of human immunodeficiency virus (HIV) infection, it is currently on the rise in the homosexual community due to a relaxation of prevention. This infectious venereal disease can affect all tissues and organs. If left untreated, syphilis progresses through four stages: primary, secondary, latent, and tertiary. Syphilitic hepatitis (SH) is one of the less common manifestations of the secondary stage. Syphilis remains one of the poorly understood etiologies of liver dysfunction. Rapid treatment and effective control of the disease are only possible with early diagnosis. We report a rare and unusual case of a young man with a history of multiple sexual partners who developed acute hepatitis. After a comprehensive biological and morphological investigation, he was diagnosed with secondary syphilitic hepatitis and was treated with ceftriaxone with spectacular clinical and biological improvement.

## Patient and observation

**Patient information:** a 25-year-old male patient, with a medical history of multiple sexual partners and controlled asthma, without any history of toxic substances, alcohol, or medications. He presented 2 months before his admission, with a gradual onset isolated hepatic colic; 6 weeks after his initial presentation, he developed a cholestatic jaundice with pruritus. He had no fever, fatigue, anorexia, or waist pain.

**Clinical examination:** the general examination found a patient in a stable neurological state, which presented an intense mucocutaneous icterus, and lesions of scrapings. The abdominal examination showed no obvious signs of clinical portal hypertension (PH) and the liver examination was normal. The lymph node assessment revealed multiple lymph nodes: 2 in the submandibular area, 2 in the occipital area, and one in the epitrochlear area. We also found bilateral inguinal adenopathies. No genital or buccal lesions were found.

**Chronology:** following a satisfactory clinical examination, the patient underwent an ultrasound scan and a biological workup, followed by an abdominal CT scan and a liver biopsy.

**Diagnostic procedure:** an abdominal and pelvic ultrasound was performed and showed hilar adenopathy, without dilatation of the intra- or extra-hepatic bile ducts, and no portal hypertension in ultrasound was found. A thoracic-abdominal-pelvic scanner showed peri-portal edema and adenopathies above and below the diaphragm without suspicious lesions of malignity. Biologically, there was acute cytolysis with aspartate aminotransferases (ASAT) at 1612 IU/L, alanine aminotransferases (ALAT) at 1506 IU/L, icteric cholestasis with alkaline phosphatases (ALP) at 305 IU/L, gamma-glutamyl transferases at 299 IU/L, and total bilirubin at 228mg/L with direct bilirubin at 181mg/L. No hepatocellular insufficiency was found. The acute viral serologies were all negative (Hepatitis A virus, Hepatitis C virus, Hepatitis E virus, Human Immunodeficiency virus, Epstein-Barr virus, Cytomegalovirus), and other autoantibodies were all negative. Given the presence of adenopathy and sexual risk factors, a syphilis serology was requested and the patient tested positive for a Treponema Pallidum Hemagglutination Assay (TPHA) at 2560UI/L, and Venereal Disease Research Laboratory (VDRL) at 1/32 UI/L. After ruling out all etiologies of cytolytic hepatitis, we retained the diagnosis of syphilitic hepatitis. He underwent liver biopsy which showed a Metavir score classified A2F2 and an inflammatory infiltrate with polynuclear cells with granulomatous hepatitis, with the presence, on immunohistochemistry, of anti-treponemal-pallidum antibodies.

**Therapeutic intervention:** taking into account the hepatic toxicity of penicillin G, an empiric treatment with ceftriaxone 2g/d was started with a spectacular biological improvement at H48 of the beginning of the treatment: decrease in AST to 816 IU/l versus 1190 IU/l and ALT to 935 IU/l versus 1155 IU/l (Table 1).

**Table 1:** liver biochemical profile of our patient before and after antimicrobial treatment

	Before treatment	H48 after treatment	7 days after treatment
ASAT (n< 34 UI/l)	1190	816	238
ALT (n < 55 UI/l)	1155	935	330
PA (n < 150 UI/l)	255	225	165
γGT (n < 33 UI/l)	231	297	231
Bilirubin e (μmol/l)	174	82	47

VDRL: venereal disease research laboratory; TPHA: treponema pallidum haemagglutination assay; AST: aspartate aminotransferase; ALT: alanine aminotransferase; γGT: gamma-glutamyl transpeptidase; PA: alkaline phosphatase.

**Follow-up and outcomes:** after 14 days of antibiotic therapy, an improvement in symptoms was observed with regression of jaundice and a significant reduction in transaminases. The patient also reported an improvement in their quality of life. Follow-up examinations included a non-specific liver function test and syphilis serologies: TPHA, and VDRL. There was no major adverse or unexpected events reported during the follow-up.

**Patient´s perspective:** “when I received my initial diagnosis, I was very worried. However, the medical team explained the treatment options to me well and supported me throughout the process. With the treatment, I was able to see significant improvements in my health. The regular follow-ups and encouragement from my doctors helped me a lot. I am grateful for the care and attention I received.”

**Patient consent:** written informed consent has been obtained from the patient for the publication of this case report.

## Discussion

The first association between hepatitis and syphilis was described in 1943 by Hahn. More than 100 cases have been described worldwide [1], syphilitic hepatitis has a variable degree of incidence from 0.24% to 17%, with the development of clinically evident hepatitis being quite rare [2]. It is characterized by a significant increase in alkaline phosphatase, in contrast to a slight increase in aminotransferase levels, rarely leading to an increase in bilirubin levels. A disproportionate increase in alkaline phosphatase levels, sometimes 10 times the normal level, in the absence of cholestasis. In 2004, Mullick recommended the following criteria for syphilitic hepatitis: abnormal liver enzyme levels indicative of liver damage, serological evidence of syphilis with a positive TPHA titer in conjunction with an acute clinical presentation consistent with secondary syphilis, exclusion of other causes of liver damage, and improvement in liver enzyme levels with appropriate antimicrobial therapy has been suggested as a hallmark of syphilitic hepatitis.

Syphilis is usually considered in the differential diagnosis of genital ulcers, generalized lymphadenopathy, and various mucocutaneous manifestations, especially in high-risk individuals. Most reviews report skin involvement in about 90% of cases [3] and abnormal liver enzyme levels in about 10% of cases. In rare cases, clinical hepatitis with skin manifestations has also been described. Numerous cases of syphilitic hepatitis have also been reported in HIV-infected patients. Treatment of syphilis results in rapid resolution of biochemical abnormalities [4]. A review conducted of 73 articles containing 144 cases of HS in adults, showed that liver lesions usually occurred at the onset of syphilis, which was easily missed as a diagnosis due to the nonspecific nature of the presenting symptoms. The results also revealed that HS was more often diagnosed in men [5]. The clinical manifestations of HS in adults are often nonspecific and multifaceted. Rash, fatigue or lack of appetite, hepatomegaly, and jaundice were the most common. Rashes are often presented as multiple, nonpruritic, erythematous, nonconfluent maculopapular lesions, usually concentrated on the trunk, palms, and soles.

Hepatosplenomegaly is often found on physical examination or imaging. Laboratory tests of HS will show abnormal liver enzymes with markedly increased ALP and GGT, in contrast to mildly elevated ALT or AST levels. Histologic features of HS include inflammatory infiltration of the bile ducts, which may be related to the elevated blood levels of ALP and GGT. Hepatic granuloma is another feature of HS [6]. It is very difficult to identify spirochetes in liver tissue in these patients. Only 19 patients had spirochetes in the liver tissue on immunohistochemical staining [7] or Warthin-Starry staining [8].

The liver biopsy usually shows focal hepatocyte necrosis, noncaseating granulomas, and portal tracts with a mixed inflammatory infiltrate [9]. It is important to note that immunohistochemical staining detects spirochetes in only up to 50% of patients and, therefore, has low sensitivity for diagnosing syphilitic hepatitis [10]. The presence of spirochetes is rare but confirmatory. Penicillin is the treatment of choice for all stages of syphilis.

Our patient presented with signs and symptoms of hepatitis with significant cytolysis and icteric cholestasis, the diagnosis of syphilitic hepatitis was confirmed on elevated VDRL titers, positive TPHA, no evidence of fatty changes in the liver on abdominal ultrasound, a liver biopsy was performed an inflammatory infiltrate with polynuclear cells with the presence, on immunohistochemistry, of anti-treponemal-pallidum antibodies. The involvement of syphilis as the cause of hepatitis was based on the characteristic liver enzyme profile with a significant elevation of the alkaline phosphatase level, negative serology for hepatotropic viruses and elimination of alcoholic origin, and rapid resolution of the symptoms and biochemical abnormalities with treatment.

## Conclusion

Syphilis is an unrecognized cause of hepatitis. It should be a differential diagnosis in all patients with abnormal liver biochemical marker levels, especially in patients with risky sexual behavior. Syphilitic hepatitis has been defined as the combination of elevated liver enzymes, positive syphilis serology, absence of other causes of hepatobiliary injury, and improvement in liver enzymes with appropriate antibiotic therapy. Given the morbidity and mortality associated with a missed diagnosis, an early diagnosis and appropriate management are essential. Think about syphilis, especially if there are sexual risk factors.
